# Interactive Cascaded Network for Prostate Cancer Segmentation from Multimodality MRI with Automated Quality Assessment

**DOI:** 10.3390/bioengineering11080796

**Published:** 2024-08-06

**Authors:** Weixuan Kou, Cristian Rey, Harry Marshall, Bernard Chiu

**Affiliations:** 1 Department of Electrical Engineering, City University of Hong Kong, Hong Kong; weixuakou2-c@my.cityu.edu.hk; 2 Schulich School of Medicine & Dentistry, Western University, London, ON N6A 5C1, Canada; cristian.rey@lhsc.on.ca; 3 Department of Radiology, Vanderbilt University Medical Center, Nashville, TN 37232, USA; harry.marshall@vumc.org; 4 Department of Physics & Computer Science, Wilfrid Laurier University, Waterloo, ON N2L 3C5, Canada

**Keywords:** prostate lesion segmentation, automatic quality assessment, interactive segmentation

## Abstract

The accurate segmentation of prostate cancer (PCa) from multiparametric MRI is crucial in clinical practice for guiding biopsy and treatment planning. Existing automated methods often lack the necessary accuracy and robustness in localizing PCa, whereas interactive segmentation methods, although more accurate, require user intervention on each input image, thereby limiting the cost-effectiveness of the segmentation workflow. Our innovative framework addresses the limitations of current methods by combining a coarse segmentation network, a rejection network, and an interactive deep network known as Segment Anything Model (SAM). The coarse segmentation network automatically generates initial segmentation results, which are evaluated by the rejection network to estimate their quality. Low-quality results are flagged for user interaction, with the user providing a region of interest (ROI) enclosing the lesions, whereas for high-quality results, ROIs were cropped from the automatic segmentation. Both manually and automatically defined ROIs are fed into SAM to produce the final fine segmentation. This approach significantly reduces the annotation burden and achieves substantial improvements by flagging approximately 20% of the images with the lowest quality scores for manual annotation. With only half of the images manually annotated, the final segmentation accuracy is statistically indistinguishable from that achieved using full manual annotation. Although this paper focuses on prostate lesion segmentation from multimodality MRI, the framework can be adapted to other medical image segmentation applications to improve segmentation efficiency while maintaining high accuracy standards.

## 1. Introduction

Prostate cancer (PCa) is the most common type of cancer and the second leading cause of cancer-related deaths among men in the United States, accounting for approximately 29% of new cancer cases and 11% of all cancer deaths in 2023 [1]. The five-year survival rate of PCa exceeds 97% with early diagnosis and treatment [1], highlighting the critical need for efficient and accurate PCa detection methods in clinical practice. Conventional screening methods, such as digital rectal examination (DRE) and blood tests for prostate-specific antigen (PSA), are highly sensitive but have low specificity [2], which often leads to overtreatment. After these initial screenings, the standard diagnostic procedure is a systematic transrectal ultrasound (TRUS)-guided biopsy. However, this method has several drawbacks, primarily its inability to precisely visualize and target prostate lesions. This limitation often results in missed cancers or unnecessary prostate biopsies, potentially causing unnecessary pain to patients [2].

Multiparametric magnetic resonance imaging (mpMRI) has shown superior performance in distinguishing clinically significant PCa (csPCa) from benign conditions, providing more precise PCa localization than ultrasound imaging [3,4]. Consequently, mpMRI has gained increasing attention in the detection and diagnosis of PCa. In order to standardize the use of mpMRI on prostate disease diagnosis, Prostate Imaging—Reporting and Data System (PI-RADS) [5,6,7,8] was developed as a consensus guideline, which recommends the combination of T2-weighted (T2W) MRI with functional MRI sequences, such as diffusion-weighted imaging (DWI) and dynamic contrast-enhanced (DCE) MRI, to stage and delineate PCa. Additionally, the apparent diffusion coefficient (ADC) sequence is commonly used in clinical practice and is derived from the DWI sequences of different b-values [5].

Although mpMRI is a powerful non-invasive technique for detecting PCa, and mpMRI-guided biopsies can significantly improve diagnosis accuracy while reducing unnecessary pain to patients, the analysis of mpMRI is time-consuming and subject to significant inter-observer variability due to differences in radiologist expertise. Therefore, computer-aided MRI PCa segmentation methods are highly demanded in clinical practice. Recent advancements in deep learning, particularly convolutional neural network (CNN)-based methods [9,10], have shown superior performance in medical image segmentation. However, current automated methods [9,10,11,12,13,14,15,16,17,18,19] have not attained sufficient accuracy and robustness in all cases. This limitation primarily stems from inherent challenges in prostate mpMR images, including low tissue contrast, irregular shapes, ambiguous boundaries, and large variations across patients [20].

In order to address these issues, we propose an interactive cascaded framework for PCa segmentation. The central hypothesis of our approach is that human intervention in automated segmentation approaches will provide a leap in lesion identification and segmentation accuracy. However, the need for a human observer to review all axial images and provide annotation would have been time-consuming. Our strategy for improving efficiency involves two crucial steps. First, we train a network to automate region of interest (ROI) detection, providing useful information to alleviate expert manual labor. Second, we automate the flagging of “inaccurate” ROI to avoid the need for users to manually review all results. The expert user can then focus on editing only the ROI for these inaccurate cases, thereby improving the cost-effectiveness of the review workflow. However, because the automatic evaluation of the quality of an ROI is a nontrivial problem itself, we propose a regression network we call rejection network that rates the automatically generated lesion segmentation result and flags low-quality detection results for user intervention. Users would identify a bounding box enclosing the lesion, and this bounding box would then assist another network to generate the final lesion boundary. Notably, this approach involves the delicate balance between the observer’s effort in editing the ROI and the final segmentation performance. That is, increasing the number of images edited by the expert observer will improve segmentation performance and will decrease cost-effectiveness. We conducted extensive experiments to evaluate the trade-off between accuracy and cost-effectiveness. The results we present allow users to choose a suitable cutoff point to balance the amount of user interaction and the segmentation accuracy desired for an application.

In this study, we used T2W, ADC, and DWI with a high b-value (DWI_*hb*_) for PCa segmentation, opting to exclude the DCE sequence due to its longer acquisition time and the risk of allergic reactions to intravenous contrast in clinical practice [5]. The inclusion of the DCE sequence has been reported to achieve similar diagnostic accuracy as cases without DCE [8]. In PI-RADS version 2.1, the role of DCE is limited to that of a tiebreaker between a PI-RADS score of 3 and 4 in the peripheral zone [8]. We evaluated the proposed method on two publicly available prostate datasets. The experimental results demonstrate that the proposed method outperforms existing state-of-the-art automated PCa segmentation methods and attains competitive performance with significantly fewer human interventions compared to state-of-the-art fully interactive segmentation methods.

## 2. Related Works

### 2.1. Deep Learning for Prostate Cancer Segmentation

Deep learning-based automated segmentation methods [9,10,11,12,13,14,15,16,17,18,19] have shown promise in effectively detecting and staging PCa from mpMRI by automatically learning abundant semantic features. Wang et al. [11] proposed a dual-path CNN framework that separately extracts T2W and ADC features and then fuses them to generate the final results. Instead of solely fusing features before the final classifier, Zhang et al. [16] developed a dual-path self-attention distillation network to extract attention maps for T2W and ADC features in each feature layer of the encoder and then fuse them within both the current and adjacent layers. They further used the Kullback-Leibler divergence loss to enforce agreement in T2W and ADC feature extraction at each feature level. Jiang et al. [19] proposed a three-stream fusion U-Net to separately extract T2W, ADC, and DWI features and then fuse these features in each layer of the encoder. An adaptive weight was introduced for each stream, enabling the network to automatically highlight the most relevant stream.

The above methods [11,16,19] process each MRI sequence individually and design sophisticated modules to fuse information from different streams. Isensee et al. [10] proposed nnU-Net and demonstrated that concatenating various MRI sequences along the channel dimension in the input image, combined with appropriate preprocessing, can achieve state-of-the-art performance across multiple tasks. nnU-Net is more computationally efficient than processing each sequence separately. Mehralivand et al. [17] conducted experiments to investigate PCa segmentation performance with an automated cascaded segmentation framework on biparametric MRI. They reported a mean Dice similarity coefficient (DSC) of 0.359 and identified a high number of false positives in predictions as a significant impediment to model performance. In order to address this, they trained an independent network to filter false positives in predictions, but the improvements were limited [17].

**Figure 1:**
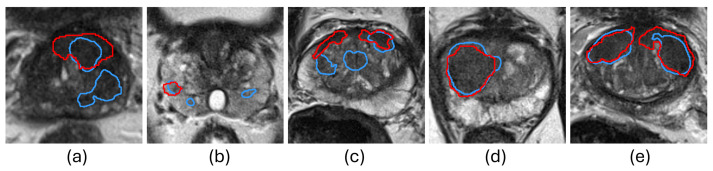
Examples of PCa segmentation results from U-Net [9]. The red and blue contours represent ground truth and algorithm segmentation, respectively. (**a**–**c**) show cases with false positives and false negatives generated by the automated segmentation method, and (**d**,**e**) show cases where the automated segmentation method demonstrates superior performance.

Reducing false positives and false negatives in predictions is the key to improving PCa segmentation performance. Integrating human expertise and automated approaches is a promising direction for achieving this. Interactive segmentation algorithms incorporate human expertise into CNN segmentation models to highlight specific regions, thereby achieving robustness and superior performance on various tasks of medical image analysis [21]. Kirillov et al. [22] proposed the Segment Anything Model (SAM) to transform the relative coordinates of user interactions into sinusoidal-like embeddings, integrating them into the decoder to enhance focus on specific regions of interest in the deep-layer features. By incorporating human interactions, SAM outperforms state-of-the-art automated methods in various tasks, demonstrating the effectiveness of human knowledge in improving the performance of CNN-based methods. However, SAM requires users to provide interactions with each input image, which limits its cost-effectiveness in medical imaging applications, where hundreds of images are required to be segmented for clinical analyses. A potential approach to improve the cost-effectiveness of SAM is to automate ROI detection but allow users to edit if the ROI is not accurate. This strategy would allow users to focus only on cases where automated segmentation methods [9,10,11,12,13,14,15,16,17,18,19,23] underperform. As illustrated in Figure 1, the automated segmentation method performs well in Figure 1d,e but fails in Figure 1a–c. If bounding boxes were automatically extracted from the results in Figure 1d,e, they could replace human inputs in SAM for efficient and accurate segmentation. On the other hand, the low-quality ROI generated for Figure 1a–c would not result in accurate segmentation by SAM. The key is in the development of an assessment workflow for automatically generated ROI that would allow for the accurate flagging of low-quality ROI for user intervention.

### 2.2. Automated Quality Assessment of Segmentation Results

Although various algorithms [9,10,17,22] have been proposed to generate segmentation results for medical images, the evaluation of segmentation quality is typically carried out manually by experts. Manually reviewing the quality of each result is impractical for the clinical implementation of these algorithms. Therefore, the automated quality assessment of segmentation results generated by CNN-based algorithms is a crucial step in streamlining user-friendly computer-aided diagnosis [24]. Jiang et al. [25] proposed an IoU-Net to regress the intersection-over-union (IoU) of the bounding box-level metrics of the predicted results. Huang et al. [26] advanced this approach by proposing the Mask Scoring R-CNN, which utilizes instance-level IoU to quantify the quality of region proposals in Mask R-CNN [27]. Eidex et al. [28] integrated a mask scoring head of [26] into their cascaded CNN to improve the performance of coarse-to-fine segmentation. Although the predicted IoU scores can approximate the actual segmentation quality, as reported in [26], these methods [25,26,28] only integrate IoU scores into the model to adaptively refine the low-quality results without investigating the potential of integrating quality scores into a human-in-the-loop segmentation. Zhou et al. [24] used a similar mask scoring head as [26] to rank the segmentation quality of axial images within a 3D volumetric image to allow user input for iterative segmentation enhancement. As their goal was just to rank the segmentation quality of axial images within a 3D volumetric image, they did not consider the problem of providing a consistent quality score across patients. A consistent assessment across patients is critical in a medical image segmentation workflow, and this is what we aim to establish in this paper.

## 3. Method

### 3.1. Overall Workflow

**Figure 2:**
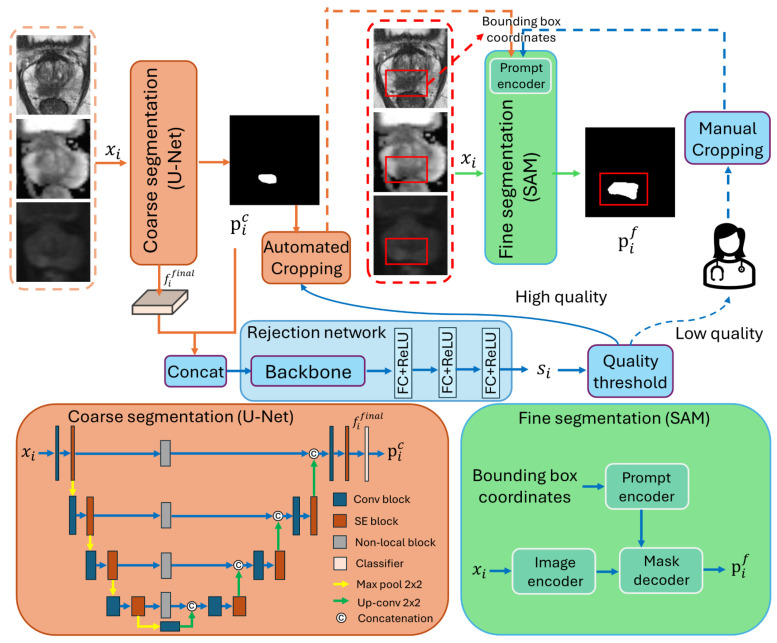
The workflow of the proposed interactive cascaded network.

The overall workflow of our proposed interactive cascaded network is illustrated in Figure 2, consisting of a coarse segmentation U-Net [9], a rejection network, and a SAM [22] to generate the final segmentation. Let $x_{i}\in R^{3\times H\times W},i\in \left\{1,2,\ldots ,N\right\}$ be an input image block with an axial spatial size of H×W, and the three channels represent the T2W, ADC, and DWI_*hb*_ image modalities. All entities with the subscript *i* in the description below are associated with the *i*th image, and *N* represents the total number of images. $x_{i}$ is first fed to the coarse segmentation U-Net, generating the prediction $p_{i}^{c}\in R^{2\times H\times W}$ as follows:

(1) $$p_{i}^{c}=\theta_{C}\left(x_{i}\right),$$

where $\theta_{C}$ represents the coarse segmentation U-Net. The two channels in $p_{i}^{c}$ provide background and foreground segmentation results. We denote $f_{i}^{final}$ as the features extracted by the model before the final classifier stage of $\theta_{C}$. The concatenation of $f_{i}^{final}$ and $p_{i}^{c}$ serves as the input of the rejection network, which can be represented by

(2) $$s_{i}=\theta_{R}\left(cat\left(f_{i}^{final},p_{i}^{c}\right)\right),\;$$

where $\theta_{R}$ represents the rejection network, and cat represents the concatenation operation. $s_{i}$ is the segmentation quality score generated by $\theta_{R}$. The Dice similarity coefficient (DSC) between the prediction $p_{i}^{c}$ and the segmentation ground truth $y_{i}$ is used as the target quality score to be estimated in our study.

Segmentation results with low $s_{i}$ are flagged for the users to input a bounding box enclosing the lesion; for segmentation results with high $s_{i}$, bounding boxes are automatically cropped from the boundary segmented by the coarse segmentation U-Net. The manually and automatically generated bounding boxes are fed to the SAM for further refinement, resulting in the final segmentation results $p_{i}^{f}$:

(3) $$p_{i}^{f}=\theta_{F}\left(x_{i},bbox\left(p_{i}^{c}|s_{i}\right)\right),\;$$

where $\theta_{F}$ indicates the fine segmentation SAM. $bbox(p_{i}^{c}|s_{i})$ represents the automatically and manually generated upper left and lower right coordinates of the bounding box derived from $p_{i}^{c}$ based on $s_{i}$.

### 3.2. Network Structure

#### 3.2.1. Coarse Segmentation Network

The coarse segmentation network has a similar structure to U-Net [9] but with the Squeeze-and-Excitation (SE) [29] and non-local blocks [30] integrated to enhance the feature representations, as shown in Figure 2. The encoder path comprises five convolutional blocks with four pooling operations, as in [9], resulting in an output stride of 16. The SE blocks perform global average pooling followed by two fully connected layers to adjust channel-wise feature scaling dynamically, whereas non-local blocks enhance the spatial attention of features in skip connections by capturing long-range dependencies in an image.

#### 3.2.2. Rejection Network

The rejection network was proposed to estimate the quality of the boundaries segmented by the coarse segmentation network so that low-quality segmentation results can be flagged for human intervention. EfficientNet-B5 [31] was used as the backbone to balance performance and computational efficiency [31]. Three fully connected (FC) layers with ReLU activations from the regression head were used to estimate the quality score of each image. We set the outputs of the first two fully connected layers of the regression head to 1024 and 512, respectively. The output of the final fully connected layers is set to 1 to output the estimated quality score.

Images with a score lower than the threshold *t* are flagged for user intervention. Users can input a bounding box enclosing the lesion. For images with a score higher than the threshold, the bounding boxes are automatically determined based on the boundary segmented by the coarse segmentation U-Net. All bounding boxes are expanded by 40%. The expansion of the automatically determined boundary boxes provides a margin of error for the coarse segmentation algorithm. For manual identification, the expansion reduces the dependence on the accuracy in the bounding box selection, which may vary across users with different levels of expertise in prostate MRI.

#### 3.2.3. Fine Segmentation Network

In our framework, both manually and automatically cropped bounding boxes can be treated as hints to guide the fine segmentation network in highlighting the specific regions. SAM [22] was used as the fine segmentation network to incorporate the generated bounding box information and enhance model performance. SAM consists of an image encoder, a prompt encoder, and a mask decoder, as detailed in [22]. We use the ViT-base model [22] as the image encoder to balance segmentation performance and computational efficiency, given that larger ViT models offer only marginal improvements in accuracy while significantly increasing computational cost [22,32]. The image encoder takes input images as inputs, while the upper left and lower right coordinates of the generated bounding box serve as inputs for the prompt encoder.

### 3.3. Model Training

The three networks involved in the proposed workflow were trained independently. The coarse segmentation U-Net is first trained with Dice loss [19], denoted by $L_{c}$:

(4) $$L_{c}=1-\frac{1}{N}\sum_{i=1}^{N}\frac{2\sum_{j=1}^{H\times W}p_{i,j}^{c}y_{i,j}}{\sum_{j=1}^{H\times W}\left(p_{i,j}^{c}+y_{i,j}\right)},$$

where both $y_{i}$ and $p_{i}^{c}$ have the same size as the input image $x_{i}$, which has a height of *H* and a width of *W*. $y_{i}$ is a binary segmentation map indicating whether each pixel of the input image is within a manually segmented lesion, whereas $p_{i}^{c}$ is the probability map generated by U-Net for each pixel being inside the lesion. $p_{i,j}^{c}$ and $y_{i,j}$ represents the $j^{th}$ pixel in $p_{i}^{c}$ and $y_{i}$, respectively. *N* represents the number of input images.

Once optimized, the parameters of the coarse segmentation U-Net were frozen, and the mean square error (MSE) loss was used to train the rejection network, denoted by $L_{r}$:

(5) $$L_{r}=\frac{1}{N}\sum_{i=1}^{N}{\left(s_{i}-d_{i}\right)}^{2},\;$$

where $d_{i}$ is the DSC calculated between $p_{i}^{c}$ and $y_{i}$, which is considered the target quality score, and $s_{i}$ is the quality score of $p_{i}^{c}$ predicted by the rejection network. *N* represents the number of input images.

SAM was initialized with the publicly available pretrained ViT-base model [22]. As the image encoder and prompt encoder were intensively trained and shown to be generalizable [22], we follow [32] in not further training them. With these encoders frozen, the decoder was fine-tuned with $L_{f}$, which is expressed below and is detailed in [32]:

(6) $$L_{f}=-\frac{1}{N}\sum_{i=1}^{N}\sum_{j=1}^{H\times W}\left[y_{i,j}\log\left(p_{i,j}^{f}\right)+\left(1-y_{i,j}\right)\log\left(1-p_{i,j}^{f}\right)\right]+\left(1-\frac{1}{N}\sum_{i=1}^{N}\frac{2\sum_{j=1}^{H\times W}p_{i,j}^{f}y_{i,j}}{\sum_{j=1}^{H\times W}\left(p_{i,j}^{f}+y_{i,j}\right)}\right),$$

where both $y_{i}$ and $p_{i}^{f}$ have the same size as the input image $x_{i}$, which has a height of *H* and a width of *W*. $p_{i}^{f}$ is the prediction of SAM, and $y_{i}$ is the corresponding ground truth. $p_{i,j}^{f}$ and $y_{i,j}$ represents the $j^{th}$ pixel in $p_{i}^{f}$ and $y_{i}$, respectively. *N* represents the number of input images. We note that the first term of $L_{f}$ is the cross-entropy loss [9], and the second term is the Dice loss [19].

## 4. Experimental Setup

### 4.1. Data

We evaluated our method on the following two publicly available datasets. The annotations for Prostate158 are publicly available, while the segmentation of the prostate lesion in PROSTATEx2 was performed by us.

**Prostate158:** This dataset comprises 158 annotated prostate MR images acquired by 3-T Siemens scanners [33]. All contributions were approved by the institutional ethics committee, as stated in [33]. The segmentation annotations of prostate zones and cancer were manually created by two board-certified radiologists [33]. The ADC and DWI sequences were resampled to the orientation and voxel spacing of the T2W sequence [33]. The voxel sizes of the T2W images vary from 0.272 mm to 0.495 mm, with a thickness from 3 mm to 3.15 mm. The T2W, ADC, and DWI images were further resampled to the median voxel spacing of 0.4×0.4×3 mm^3^. Following the data partition in [33], the dataset was split into training (119 cases), validation (20 cases), and testing (19 cases) subsets on a patient basis.

**PROSTATEx2:** The training set of PROSTATEx2 [23,34,35] was used in our study, consisting of 99 prostate MR images acquired by 3T Siemens scanners. All contributions were approved by the institutional ethics committee, as stated in [23,34,35]. The segmentation annotations of PCa were manually performed by a medical student and a board-certified radiologist. The ADC and DWI sequences were resampled to the orientation and voxel spacing of the T2W sequence, achieving a 0.5×0.5 mm in-plane resolution and 3.6 mm slice thickness. We randomly split the dataset on a patient basis into training (60 cases), validation (9 cases), and testing (30 cases) sets.

### 4.2. Evaluation Metrics

Two metrics were used to evaluate the segmentation performance. The Dice similarity coefficient (DSC) between the algorithm segmentation, *P*, and the corresponding ground truth, *G*, is defined by $DSC=\frac{2|P\cap G|}{\left|P|+|G\right|}$, where |·| denotes the area of the operand. The Hausdorff distance (HD) evaluates the maximum distance from a point from ∂P (or ∂G) to its nearest point in ∂G (or ∂P). The 95%HD defined below is a variant designed to reduce the effect of outliers:

(7) $$95\%HD={\left[\left\{\min_{j\in \partial G}d\left(i,j\right):\forall i\in \partial P\right\},\left\{\min_{i\in \partial G}d\left(i,j\right):\forall j\in \partial P\right\}\right]}_{95th}.\;$$

where d(i,j) denotes the distance between points *i* and *j*.

An R-squared value ($R^{2}$) was used to evaluate the performance of the rejection network:

(8) $$R^{2}=1-\frac{\sum_{i=1}^{N}{\left(d_{i}-s_{i}\right)}^{2}}{\sum_{i=1}^{N}{\left(d_{i}-\bar{d}\right)}^{2}},\;$$

where $s_{i}$ is the prediction of the rejection network, $d_{i}$ is the DSC ground truth, and $\bar{d}$ is the mean of $d_{i}$.

The lesion-level recall and precision defined in [19,36] were applied to evaluate the model performance in lesion localization. Each lesion was considered a 3D entity, and all metrics were calculated on a lesion basis. Each of the *I* ground truth lesion volumes ${\left\{G_{i}\right\}}_{i=1}^{I}$ is considered a true positive (TP) if the overlap with any *J* algorithm segmented lesion volumes ${\left\{P_{j}\right\}}_{j=1}^{J}$, single or multiple, is larger or equal to a threshold, τ, and a false negative (FN) otherwise (i.e., $G_{i}$ is a TP if $\sum_{j=1}^{J}|G_{i}\cap P_{j}\left|/\right|G_{i}|\geq \tau$, or a FN otherwise). The lesion-level recall (or true positive rate, TPR) is defined as TP/(TP+FN). For the calculation of precision (or positive predictive value, PPV), the definition of TP is based on the algorithm segmentations $P_{j}$. That is, $P_{j}$ is a TP if $\sum_{i=1}^{I}|G_{i}\cap P_{j}\left|/\right|P_{j}|\geq \tau$, or it is false positive (FP) otherwise. The precision is defined to be TP/(TP+FP). We used a low threshold τ=0.1 due to substantial interobserver variability in localizing lesions. Lesions can be underestimated even with manual segmentation, typically requiring margins up to 10 mm to be added to boundaries for focal treatments [37,38]. The $F_{1}$ score was calculated to summarize the lesion-level precision and recall as $F_{1}=2\cdot \frac{precision\cdot recall}{precision+recall}$.

### 4.3. Implementation Details

Our model was implemented with the PyTorch library [39] and executed on an NVIDIA RTX3090 GPU. Adam optimizer was used to optimize the coarse segmentation U-Net and the rejection network with a batch size of 16. The initial learning rate was set to 0.001 for the coarse segmentation U-Net. For the rejection network, the initial learning rate was set to 0.0001 for the backbone and 0.001 for the regression head. The learning rate was decreased according to polynomial decay with an exponent of 0.9. We followed [32] to use AdamW [40] optimizer ($\beta_{1}=0.9$, $\beta_{2}=0.999$), with an initial learning rate of 0.0001 and a weight decay of 0.01 to fine-tune the decoder part of SAM. The batch size was 4.

The coarse segmentation U-Net was trained for 200 epochs. All input images for coarse segmentation U-Net were uniformly resampled to the size of 256×256. Random horizontal flipping, rotation, and cropping were applied to augment the training set. Once optimized, the coarse-segmentation U-Net was frozen, and the rejection network was trained for 200 epochs. The optimized coarse-segmentation U-Net and rejection network were used in the inference stage.

The fine segmentation SAM was first initialized with the publicly available pretrained ViT-base model [22]. The inputs to SAM included the images and bounding boxes generated from segmentation ground truth. The bounding boxes were randomly extended from 0% to 60% and then augmented with a random perturbation of 0–20 pixels in the training stage. All input images for SAM were uniformly resampled to the size of 256×256. The decoder part of SAM was fine-tuned for 150 epochs [32].

The model with the highest DSC or the lowest MSE on the validation set was used for evaluation on the test set. The intensity of T2W, ADC, and DWI_*hb*_ was separately scaled into [0,1] and normalized. The threshold, *t*, in the rejection network is a flexible parameter that can be adjusted based on the practical requirements and model performance of the coarse segmentation network. In our study, we set t=0.4 to show the model performance with a low burden of human intervention and t=0.7 for a high burden of human intervention. Section 5.2 details the influence of *t* on the model performance and burdens of human intervention for PCa segmentation.

## 5. Experimental Results

### 5.1. Comparison with State-of-the-Art Methods

The segmentation performance of the proposed interactive cascaded network was compared with the following state-of-the-art methods: nnU-Net [10], UNETR [41], MIDeepSeg [42], and SAM [22]. Specifically, nnU-Net and UNETR serve as benchmarks for automatic segmentation methods without human interventions. MIDeepSeg and SAM are fully interactive segmentation methods incorporating human interventions in each input image, serving as the upper bound of the interactive segmentation methods. In our study, we use bounding box information as the user input of SAM since it has demonstrated superior performance on medical images [32]. The manually identified bounding box inputs for SAM were simulated by expanding the bounding box enclosing manual segmentations by 40%. The same strategy was used to simulate the manual boundary box in our approach for images with coarse segmentation quality scores lower than the threshold *t*. Bounding boxes are not applicable in MIDeepSeg [42], which requires extreme points on the boundaries of target regions to generate exponential geodesic distance maps. Hence, we follow the implementation in [42] to use extreme points as user inputs and set the number of extreme points as 4. All results are calculated on a per-image basis. Paired t-tests [43] were used to evaluate the statistical significance of performance difference in terms of DSC and 95% HD.

#### 5.1.1. Comparison with Automated Segmentation Methods

Table 1 shows the DSC and 95% HD of the proposed method and the state-of-the-art automated segmentation methods nnU-Net [10] and UNETR [41] on the Prostate158 and PROSTATEx2 datasets. Model performance with t=0.4 presents scenarios where a low burden of human intervention is required, as detailed in Section 5.2. At t=0.4, 16.3% of the images from the Prostate158 test set and 20.9% from the PROSTATEx2 test set were flagged by the rejection network for user ROI identification. With manual ROI identification on approximately 20% of the images, the segmentation performance was significantly higher than automated segmentation methods (DSC: *p* = $1.8\times 10^{-8}$ for nnU-Net and $3.7\times 10^{-7}$ for UNETR in Prostate158, and *p* = $9.4\times 10^{-14}$ for nnU-Net and $3.0\times 10^{-13}$ for UNETR in PROSTATEx2; 95% HD: *p* = $7.6\times 10^{-4}$ for nnU-Net and $4.3\times 10^{-9}$ for UNETR in Prostate158, and *p* = $2.7\times 10^{-4}$ for UNETR in PROSTATEx2).

**Figure 3:**
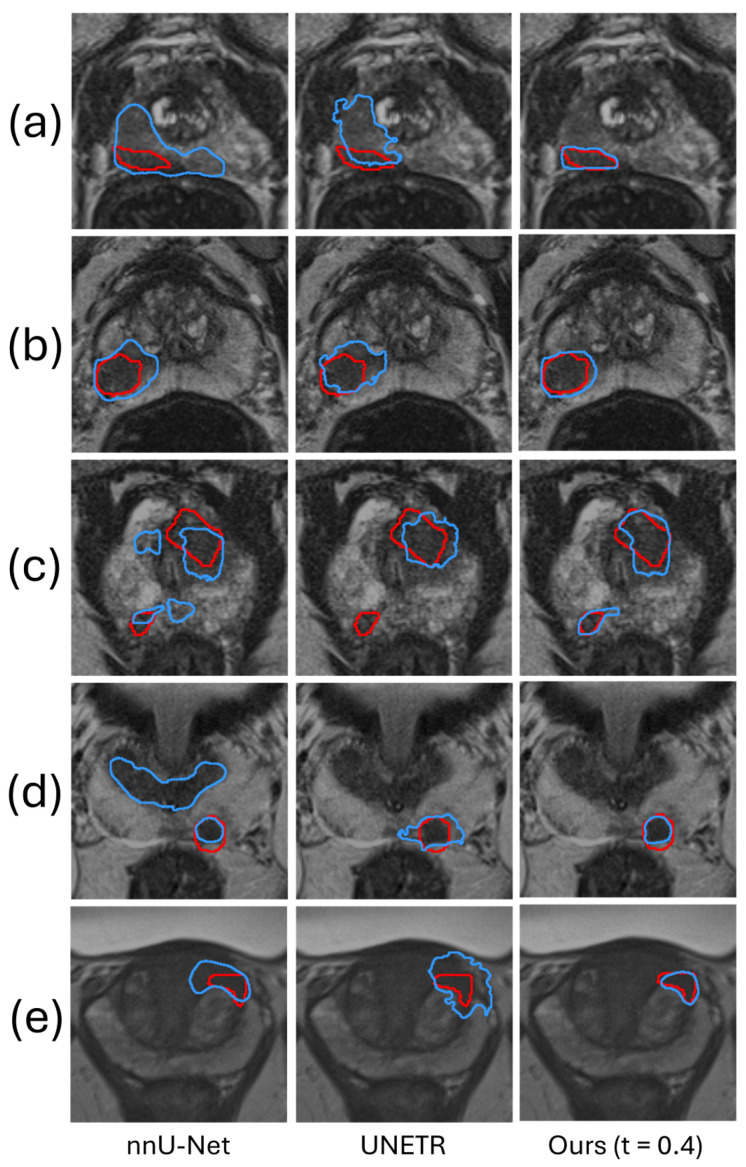
The segmentation results of our method are compared qualitatively with those produced by two automatic segmentation methods in five example images. The red and blue contours represent ground truth and algorithm segmentation, respectively. Prostate (**a**–**c**) are from the Prostate158 dataset, and Prostate (**d**,**e**) are from the PROSTATEx2 dataset.

Figure 3 shows a qualitative comparison, demonstrating the superior performance of the proposed framework. Figure 3c is a representative prostate image where nnU-Net and UNETR generated FP and FN predictions. For this example, the ROI was automatically generated by the coarse segmentation U-Net.

#### 5.1.2. Comparison with Fully Interactive Segmentation Methods

**Figure 4:**
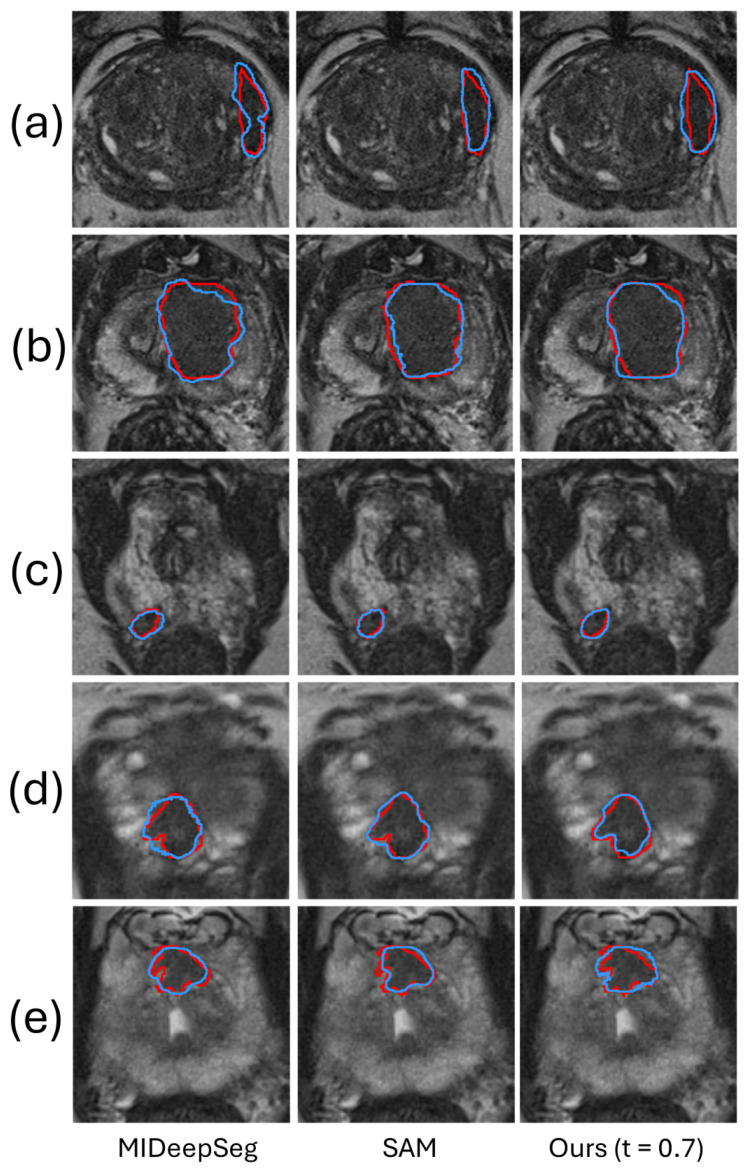
The segmentation results of our method are compared qualitatively with those produced by two fully interactive segmentation methods in five example images. The red and blue contours represent ground truth and algorithm segmentation, respectively. Prostate (**a**–**c**) are from the Prostate158 dataset, and Prostate (**d**,**e**) are from the PROSTATEx2 dataset.

Table 1 shows the DSC and 95% HD of the proposed method compared with the state-of-the-art fully interactive segmentation methods MIDeepSeg [42] and SAM [22] on the Prostate158 and PROSTATEx2 datasets. The performance by MIDeepSeg [42] and SAM [22] serves as the upper bound to assess the performance of our proposed framework with *t* set to 0.7, where images with a predicted DSC of less than 0.7 were provided a manually identified ROI. At t=0.7, 46.9% of the images from the Prostate158 test set and 51.4% from the PROSTATEx2 test set were automatically flagged by the rejection network for user interaction. In this threshold setting, the burden of human intervention for our method was half of that involved in MIDeepSeg and SAM, which require user interaction for all images. Table 1 shows that SAM has the best segmentation performance on both datasets. In comparison, our proposed framework attains competitive performance with no significant statistical differences (DSC: *p* = 0.075 in Prostate158 and 0.058 in PROSTATEx2, 95% HD: *p* = 0.49 in Prostate158 and 0.06 in PROSTATEx2), but with approximately half the human intervention burden. Figure 4 shows a qualitative comparison in five example images, demonstrating that, with ROI manually identified in half of the images in the test set, our algorithm has a segmentation performance similar to the two methods requiring interactions in all images.

#### 5.1.3. Lesion-Level Analysis

Table 1 reports the TPR and PPV of different methods [10,22,41,42], as an assessment of lesion-level false positives (FPs) and false negatives (FNs). A higher recall indicates fewer FNs, while a higher precision indicates fewer FPs. The results in the first and second rows show that high FP is the major issue in automated segmentation methods, and incorporating human intervention substantially reduces both FP and FN (third and fourth rows). The performance of our method with t=0 demonstrates that the FP and FN generated by the coarse segmentation can significantly affect the performance of the subsequent SAM. The TPR and PPV substantially improve with different levels of intervention, as shown in the results for the settings t=0.4 and t=0.7.

### 5.2. Analysis of Rejection Ratio

**Figure 5:**
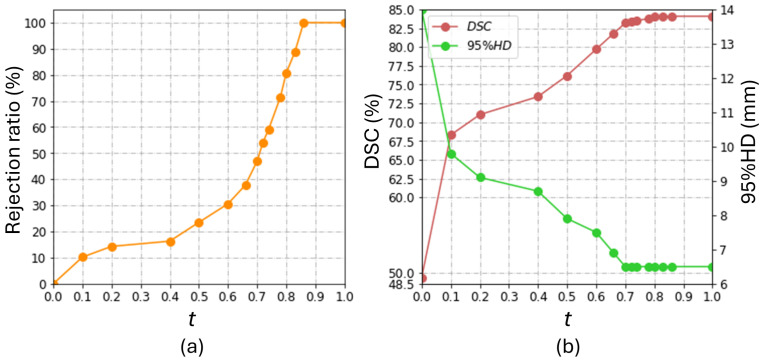
The influence of the burdens of human intervention on the model performance in the Prostate158 dataset. The rejection ratio quantifies the burden of human intervention. (**a**) shows the correlation between the threshold, *t*, and the rejection ratio in our study; (**b**) shows the effect of t on the overall model performance.

In our study, the $R^{2}$ value of the proposed rejection network is 0.75 in the Prostate158 and 0.71 in the PROSTATEx2. In order to quantify the workload required for manual ROI identification, we computed the percentage of the images requiring ROI identification, which we call the percentage rejection ratio. The rejection ratio is related to the threshold *t*, according to Figure 5a. It can be observed that the rejection ratio did not increase much when *t* was increased from 0.1 to 0.4. At this range, user involvement was less than 20%. For this reason, we used t=0.4 to represent a low level of user involvement in Section 5.1. At t=0.7, user involvement in ROI identification is approximately 50%. We used this level to represent a high level of user involvement in our comparisons in Section 5.1.

Figure 5b shows the overall performance at different levels of rejection ratio in the Prostate158 dataset. This figure shows that DSC and 95% HD enter a plateau with t>0.7. We attribute this observation to the superior performance of the coarse segmentation network on images with t>0.7, where the automatically generated bounding boxes are comparable to human inputs. Meanwhile, Figure 5a shows that the rejection ratio (i.e., the workload involved in ROI identification) drastically increases with *t* exceeding 0.7. These observations suggest that it is not cost-effective to set *t* to be higher than 0.7.

The coarse segmentation U-Net attained a DSC of 47.8 ± 26.7% and a 95% HD of 13.1 ± 10.3 mm on the Prostate158 dataset. When the bounding box information was directly generated from the coarse segmentation results without the rejection network (i.e., t=0), the DSC is 49.3 ± 28%, and the 95% HD is 14.0 ± 12.1 mm. There was limited improvement in terms of DSC and even degradation in terms of 95% HD compared to the coarse segmentation results (DSC: *p* = $9.3\times 10^{-3}$; 95% HD: *p* = $1.2\times 10^{-3}$). In contrast, the segmentation performance significantly improved even with t=0.1, which involved introducing human interactions into 10.2% of the images, as shown in Figure 5. The comparison between the performance at t=0 and t=0.1 (DSC: *p* = $6.3\times 10^{-6}$; 95% HD: *p* = $4.1\times 10^{-8}$) suggests that low-quality ROI substantially affects the segmentation accuracy from SAM. The segmentation performance at t=0.7, with 46.9% of the images provided with manually annotated ROIs, was not significantly different from that at t=1 that requires manual annotated ROI for all images (DSC: *p* = 0.075; 95% HD: *p* = 0.49). This is a key result that demonstrates the contribution of the proposed rejection network in improving the cost-effectiveness of the segmentation workflow.

### 5.3. Influence of Different Bounding Box Extension Ratio

We introduced the bounding box extension ratio to simulate scenarios where PCa ROIs may not be identified accurately due to the variability of the expertise across observers. Table 2 shows the influence of the bounding box extension ratio on model performance, with results obtained using t=0.4. The DSC and 95% HD degraded slightly when the bounding box extension ratio increased from 10% to 40%, but the difference was not statistically significant (DSC: *p* = 0.14; 95% HD: *p* = 0.47). Additionally, the lesion-level metrics remain unchanged when the bounding box extension ratio is lower than 60%, indicating that no additional false positives or false negatives are generated, with the expansion increasing from 10% to 60%. Therefore, we set the bounding box extension ratio to 40% in our experiments to maintain flexibility and model performance.

### 5.4. Training and Inference Time

We trained all methods listed in Section 5.1 on an NVIDIA RTX3090 GPU with 24 GB memory. Table 3 shows the total training time (h) and average inference time per image (ms) of different methods [10,22,41,42]. Notably, the training time of nnU-Net is significantly longer due to the default configurations for generating ensemble predictions. We trained only the decoder of SAM, as noted in Section 3.3, and as such, the training time reported includes the time involved in decoder training only. The training time of our proposed method is the sum of the training time for U-Net (1.8 h), the rejection network (6.5 h), and SAM (15.8 h). The user interpretation time is not included in the presented results, which constitutes the majority of the time cost in actual implementation. Although the average inference time of our method is longer than that of MIDeepSeg [42] and SAM [22], the difference is negligible compared to the user interpretation time. The time for radiologists to interpret PCa from mpMRI varies depending on the complexity of the case and the experience of the radiologist. Studies reported that the average interpretation time per image ranges from 1 to 4 min, and it may be even longer in clinical practice [44,45]. The inference time of our proposed method is negligible compared to clinical interpretation time. A major advantage of our approach is that human interpretation is only needed for images with low-quality U-Net segmentation, thereby allowing an expert observer to focus only on problematic cases and expedite the review. In our study, the proposed framework attained competitive segmentation performance with no significant difference compared to MIDeepSeg and SAM by incorporating human annotation in half of the images.

## 6. Discussion

We developed an interactive cascaded framework to enhance PCa segmentation performance from mpMRI and improve the efficiency of human-in-the-loop segmentation. Although networks, such as MIDeepSeg [42] and SAM [22], have been proposed to involve human interaction to improve the segmentation performance of automatic segmentation networks, the need for providing annotations in all input images limits the cost-effectiveness of these approaches. The proposed framework streamlines the interactive workflow by integrating (a) the coarse segmentation U-Net to automatically generate an initial ROI, (b) the rejection network that automatically estimates the quality of initial segmentation results and flags low-quality results for the manual identification of an ROI and (c) SAM that generated the final segmentation based on the ROI generated either manually or automatically. We conducted extensive experiments to show the contribution of the proposed rejection network and the trade-off between accuracy and cost-effectiveness. The proposed framework has demonstrated superior performance in terms of DSC and 95% HD in two publicly available datasets with manually identified ROI only for a fraction of the images.

The performance of the proposed framework benefits from the rejection network, which leverages the strengths of both automated and interactive segmentation methods. Previous studies [25,26,28] mainly focused on automatically refining low-quality results using estimated quality scores but did not integrate these scores into a human-in-the-loop segmentation. Our framework provides a method to incorporate quality score estimation into interactive segmentation, enhancing the efficiency of human-in-the-loop segmentation. The proposed rejection network improves the model performance in two ways. First, it rates and flags results where the coarse segmentation network underperforms for human intervention, thereby improving the final segmentation. Second, it enables users to focus only on low-quality results, increasing the efficiency of human-in-the-loop segmentation. At t=0.4, with 16.3% of the images from Prostate158 and 20.9% from PROSTATEx2 provided with manual intervention, the DSC increased by 24.1% and the 95% HD by 5.3 mm compared to t=0 (i.e., no user intervention) for the Prostate158 dataset (DSC: *p* = $9.4\times 10^{-8}$; 95% HD: *p* = $1.0\times 10^{-10}$). Similarly, there was a 26.8% increase in DSC and a 2.7 mm improvement in 95% HD for the PROSTATEx2 dataset at t=0.4 compared with t=0 (DSC: *p* = $5.8\times 10^{-15}$; 95% HD: *p* = $3.9\times 10^{-5}$). At t=0.7, with 46.9% of the images from Prostate158 and 51.4% from PROSTATEx2 provided manual annotations, our proposed framework attained a DSC and a 95% HD that were not significantly different from those generated by SAM with all images annotated (i.e., at t=1) (DSC: *p* = 0.075 in Prostate158 and 0.058 in PROSTATEx2; 95% HD: *p* = 0.49 in Prostate158 and 0.06 in PROSTATEx2). Based on our experimental results, we would suggest using t=0.7 to flag low-quality results for an optimal balance of segmentation performance and cost-effectiveness. In clinical practice, the selection of *t* depends on various factors, including the time users can afford, their level of expertise, and the required accuracy. A lower *t* corresponds to fewer interventions and lower segmentation performance, yet human interventions in a small number of cases can still significantly enhance model performance compared to automated methods. Users with a lower level of expertise could set a lower *t* to focus on cases for which the coarse segmentation network generates low-quality segmentation results and leave the cases with medium segmentation alone if he/she is not confident in improving the automatically generated ROI. The required accuracy of the task is the most important factor in deciding the degree of user involvement. *t* should be set higher if the task requires high accuracy. Additionally, users can choose to identify ROI at locations important for cancer diagnosis instead of identifying the ROI for all images with *t* smaller than the threshold.

Although our method has achieved high segmentation accuracy, our slice-based framework does not consider the continuity of adjacent axial slices. However, evidence suggests that 3D CNNs perform worse than 2D CNNs when slice thickness is substantially larger than in-plane voxel sizes due possibly to the limited continuity, such as in the prostate dataset in the decathlon challenge reported in [10]. Although an investigation in the current dataset is warranted, this issue is potentially present in our dataset due to similar thicknesses in mpMRI images investigated in this study. Additionally, 2D CNNs are less computationally demanding and offer more flexibility in clinical settings. For these reasons, we opted to implement and evaluate the proposed framework in a 2D setting in the current study. Future studies should investigate whether a 3D implementation, with the rejection network providing a quality score for each slice in a 3D volume, could enhance segmentation accuracy.

The proposed method only generates ROI bounding boxes for interactive segmentation. Further development of the algorithm is needed to support other types of interactions, such as clicks, scribbles, and masks. The segmentation performance associated with these interaction types is required to be thoroughly assessed in future studies.

## 7. Conclusions

We have developed a robust framework that effectively integrates automatic segmentation with user interaction to enhance the performance and cost-effectiveness of image segmentation tasks. While existing networks such as MIDeepSeg [42] and SAM [22] have incorporated human interaction to improve the accuracy of automatic segmentation, they are limited by the necessity of providing annotations for all input images, which diminishes their cost-effectiveness. Our primary innovation addresses this limitation by feeding an ROI, automatically extracted by a coarse segmentation network, into an interactive deep network to generate the final segmentation. However, because the automatic segmentation network has a limited ability to localize small and irregular-shaped lesions correctly, we proposed a rejection network to flag low-quality boundaries segmented by the coarse segmentation network for user interaction. We found substantial improvement by flagging about 20% of the images with the lowest quality score for manual annotation. With approximately 50% of the images manually annotated, the final segmentation accuracy was statistically indistinguishable from that achieved with full manual annotation. While this paper demonstrates our approach in prostate lesion segmentation from multimodality MRI, we believe it could be adapted to improve segmentation efficiency and maintain high accuracy standards in other medical image segmentation applications. Future evaluations involving image benchmarks of different organs are needed to assess the generalizability of the proposed framework.

## Figures and Tables

**Table 1:** Quantitative comparison with different methods on Prostate158 and PROSTATEx2 datasets in terms of slice-level DSC (%) and 95% HD (mm), and lesion-level recall (true positive rate, TPR, %), precision (positive predictive value, PPV, %) and F_1_ score (%). The best results are highlighted in bold.

Methods	Prostate158					PROSTATEx2				
	DSC	95% HD	TPR	PPV	F_1_	DSC	95% HD	TPR	PPV	F_1_
nnU-Net	49.5 ± 32.4	10.4 ± 8.5	70.8	71.0	70.9	43.7 ± 34.3	12.6 ± 12.7	64.5	63.6	64.0
UNETR	51.0 ± 26.1	14.3 ± 10.7	75.0	42.1	55.1	44.3 ± 26.5	15.9 ± 10.6	71.0	49.4	58.3
MIDeepSeg	80.5 ± 10.6	8.2 ± 3.5	83.3	86.3	84.8	79.2 ± 11.9	**3.0 ± 2.2**	93.5	89.7	91.6
SAM	**84.1 ± 8.6**	**6.5 ± 4.5**	**91.7**	**93.5**	**92.6**	**81.9 ± 10.7**	10.8 ± 6.7	**93.5**	**90.9**	**92.2**
ours (t = 0)	49.3 ± 28.4	14.0 ± 12.1	62.5	46.9	53.6	42.5 ± 33.2	16.7 ± 14.3	51.6	44.8	48.0
ours (t = 0.4)	73.4 ± 16.2	8.7 ± 7.5	83.3	72.9	77.8	69.3 ± 21.4	14.0 ± 7.9	90.3	70.1	78.9
ours (t = 0.7)	83.2 ± 8.9	6.5 ± 4.6	**91.7**	91.8	91.7	81.0 ± 11.6	11.2 ± 7.0	**93.5**	88.3	90.8

**Table 2:** Ablation study investigating the influence of bounding box extension ratio on the model performance in the Prostate158 dataset in terms of slice-level DSC (%) and 95% HD (mm), and lesion-level recall (True positive rate, TPR, %), precision (Positive predictive value, PPV, %) and F_1_ score (%).

Bounding Box Extension Ratio	DSC	95% HD	TPR	PPV	F_1_
10%	74.0 ± 16.0	8.5 ± 7.4	83.3	72.9	77.8
20%	73.9 ± 16.0	8.5 ± 7.5	83.3	72.9	77.8
30%	73.6 ± 16.2	8.7 ± 7.5	83.3	72.9	77.8
40%	73.4 ± 16.2	8.7 ± 7.5	83.3	72.9	77.8
50%	72.7 ± 16.4	8.8 ± 7.5	83.3	72.9	77.8
60%	71.8 ± 16.7	9.0 ± 7.8	83.3	72.9	77.8
80%	69.4 ± 17.2	9.4 ± 8.0	83.3	71.2	76.8

**Table 3:** The training and inference time of different methods. The user interpretation time is not included in the inference time reported for MIDeepSeg [42], SAM [22], and our proposed method.

Methods	Training Time (h)	Inference Time (Ms/Image)
nnU-Net	101.5	91.7
UNETR	14.6	24.4
MIDeepSeg	18.2	20.3
SAM	15.8	140.5
ours	1.8 + 6.5 + 15.8	175.7

## Data Availability

The data present in this study are publicly available in https://zenodo.org/records/6481141, https://zenodo.org/records/6592345, https://www.aapm.org/GrandChallenge/PROSTATEx-2 (accessed on 1 August 2024), reference number [23,33,34,35]. Manual annotations of lesions from the Prostatex2 database are available from the corresponding author on reasonable request.
